# New sparse implantation technique of I-125 low-dose-rate brachytherapy using concomitant short-term hormonal treatment for low and intermediate-risk prostate cancer: An initial study of therapeutic feasibility

**DOI:** 10.1038/s41598-019-55317-1

**Published:** 2019-12-10

**Authors:** Young Dong Yu, Jong Jin Oh, Hyun Soo Shin, Dong Soo Park

**Affiliations:** 1 Department of Urology, CHA Medical University, College of Medicine, Bundang CHA Hospital, Seongnam, Korea; 2 Department of Urology, Seoul National University Bundang Hospital, Seongnam, Korea; 3 Department of Radiation Oncology CHA Medical University, College of Medicine, Bundang CHA Hospital, Seongnam, Korea

**Keywords:** Oncology, Surgical oncology

## Abstract

This study aimed to evaluate the oncological outcomes and post-implantation complications of the concurrent androgen deprivation therapy (ADT) with I-125 low-dose-rate (LDR)-prostate brachytherapy (sparse implantation technique: SIT) in comparison with the conventional non-ADT using whole gland brachytherapy (CWT). 302 localized prostate cancer (PCa) patients were treated with CWT (implantation dose: 145 Gy) and 215 patients were treated with SIT, which applied reduced implantation dose of 123.5 Gy. SIT group had ADT consisting of bicalutamide 50 mg/day plus 3-month depot (11.25 mg) of leuprolide acetate subcutaneously on the post-implantation day-0. Post-implantation complications and biochemical-recurrence-free-survival (BCRS) were compared between the two groups. After ADT, SIT group had 40.9% patients (40.9%) with prostate volume reduction between 20–30%. At 3-months post-implantation, SIT group presented significantly better IPSS than CWT group (p = 0.038). Both groups showed decrease in IIEF-5 score at 3-months post-implantation, but ST group showed significantly better mean IIEF-5 scores (13.5) than the CWT group (11.1) (p = 0.045). For 3-months post-implantation dosimetry, both groups showed no significant differences regarding D90 (CWT 156 Gy vs. SIT 152 Gy). CWT group had 3 patients with rectal toxicity ≥radiation therapy oncology group (RTOG) grade 2 and 1 patient with urinary toxicity ≥RTOG grade 2 whereas SIT group had no patient with urinary or rectal toxicity ≥RTOG grade 2. Kaplan-Meier analyses showed no significant differences regarding PCSS were observed between the two groups (p = 0.350). The SIT group showed compatible oncological outcomes to the CWT and relatively smaller number of post-implantation complications within low- and intermediate-risk PCa patients.

## Introduction

The conventional treatment of localized prostate cancer (PCa) consists of radical prostatectomy (RP) and radiation therapy (RTx). Among RTx, whole gland targeted brachytherapy has shown compatible oncological outcomes with relatively low rates of postoperative complications compared to other therapeutic modalities, such as open or robotic RP^1^. Regarding brachytherapy, recent dramatic developments in imaging and biopsy tools allowed focal brachytherapy, which could reduce the postoperative complications of whole-gland treatments including genitourinary and rectal adverse event. However, focal brachytherapy carries a few limitations in its nature, including technical difficulties of precisely localizing the target volume, and post-implantation monitoring of the non-implanted prostate portions^2,3^. Furthermore, some previous studies presented that PCa consists of multifocal carcinogenic processes^4^, and the multifocality of cancer commonly accompanies small secondary lesions with low grade cellularity that were presented in several postmortem studies, or prostatectomy series^5^ Particularly, no widely accepted implantation technique for focal prostate brachytherapy has been established through previous studies.

Regarding implantation methods, prostate glands with a volum >60cc are technically more challenged for seed implantation due to an increased number of radioactive seeds required and anatomical constraints produced by pubic arch bone interference^6^. Thus, many previous studies investigated the use of androgen deprivation therapy (ADT) for prostate volume reduction before brachytherapy. However, to our knowledge, not many studies have researched the simultaneous application of brachytherapy in conjunction with ADT regarding post-implantation complications. Especially, the optimal duration of ADT has not been determined through previous literatures. According to several previous studies, including Kucway*et al*.^7^, prostate volume reduction ranges from 30% to 55% after ADT with a duration range of 1–10 months. Moreover, we speculated that the prescription dose and number of radioactive seeds required would be decreased if ADT is performed during peri-implantation period, as the prostate would shrink to a smaller size due to ADT. Consequently, we postulated that lower prescription dose accompanied by concurrent ADT would result in less complications produced by radiation. Therefore, for the purpose of reducing radiation induced complications, this study analyzed the short-term oncological outcomes and post-implantation complications of the concurrent ADT with low-dose-rate (LDR)-brachytherapy in comparison with the conventional non-ADT using whole gland brachytherapy.

## Methods

### Study design and cohort selection

Among 1,308standard 12-cores trans-rectal ultrasound (TRUS) guided biopsy proven PCa patients, 573 patients underwent LDR-brachytherapy at our institution for the treatment of clinically localized PCa between January 2010 and December 2016. After obtaining approval from the Bundang CHA Hospital Institutional Review Board (2018–09–030),the medical records of those 573 LDR-patients were retrospectively reviewed. All participants of the study provided written informed consent regarding the use of their medical records before inclusion to the present study. All study procedures including data collection and management were performed in accordance with relevant guidelines and regulations. Cohort exclusion criteria for the study were as follows; clinical tumor stage (cT) ≥cT3a, biopsy Gleason score (GS) ≥4 + 4, initial prostate specific antigen (PSA) ≥20 ng/mL, ≥6 positive cores from a single prostate lobe, history of 5-alpha reductase inhibitor use before implantation, and history of previous pelvic RTx. After applying the corresponding exclusion criteria, overall 517 patients were included in the final analysis.

### Dose planning and seed implantation

Regarding implantation techniques, two implantation modalities were performed in the current study. One is the conventional whole gland implantation technique (CWT) and total 302 patients were treated with the CWT. The CWT had the implantation dose of 145 Gy, which incorporated 75–83 I-125 seeds containing a median seed activity of 1.413 MBq (0.382 mCi). The brachytherapy planning treatment volume (PTV) of the CWT encompassed the entire prostate gland with a 5 mm periprostatic margin and the proximal 5 mm of the seminal vesicles. The other implantation technique consists of the whole gland implantation with concurrent adjuvant ADT. As many previous studies including Lee *et al*. described, average of 40% prostate volume shrinkage occurs after 3 months of ADT^8^. We speculated that concurrent use of LDR-brachytherapy with ADT would promote decreased prescription dose and smaller number of seeds than non-ADT using brachytherapy, while achieving increased malignant cell deaths. We also postulated that a decrease in overall implantation dose would eventually reduce the rate and intensity of radiation induced complications. To avoid strong radiation influence to rectum and urethra while maintaining sufficient dose coverage with adequate inter-seed spacing, radioactivity decay curve (evaluation formula: A = A0e^-(0.693t/T(1/2))^) and I-125 seed nomogram for prostate volume were plotted. By using the corresponding evaluations, 123.5 Gy of the optimal implantation dose for the concurrent ADT plus brachytherapy was obtained and this enabled a greater inter-seed spacing than the CWT. Due to a greater inter-seed spacing, we named the modified whole-gland technique as the sparse implantation technique (SIT). PTV and total radioactive source strength density were essentially the same in both implantation modalities. Among the 517 LDR-brachytherapy patients included in the study, 215 patients were treated with the SIT, and the SIT group had 55–60 seeds implanted.

The first process of both CWT and SIT was analyzing the intra-prostatic tumor volume and region by taking pre-implantation multi-parametric magnetic resonance image (mpMRI) and computed tomography (CT). All study cohort underwent T2-weighted, functional diffusion-weighted (DWI) MRI, and dynamic contrast-enhancement (DCE). MpMRI protocol sequences followed the European Society of Urogenital Radiology guidelines^9^. For MRIs performed between 2013 and 2016, Prostate Imaging Reporting and Data System (PI-RADS) version 1 was used for the evaluation of PCa, and PI-RADS version 2 was applied for the MRIs taken since 2017. A Digital Imaging and Communications in Medicine (DICOM) archive was used for matching the positions of the pathologically proven cores in relation to the target prostate tumor.

A single senior uro-radiologist performed implantation and a senior radiation oncologist as well as two experienced radiology physicists carried out pre-, intra- and post-operative dosimetry procedures.

The seed implantation procedures are primarily the same between the CWT and SIT, which are described as follows. First, under epidural anesthesia, the patient was placed at the lithotomy position with a urethral catheter inserted, and the Koelis system was applied for matching 3D-TRUS images with mpMRI outlining pathologically confirmed tumor within prostate. The tumor location was marked with highly echogenic straight shape 0.8 mm polypropylene marker (Source Link, Bard Medical, Covington, GA). This tumor location for land marking was chosen when it was markedly visible in both CT and mpMRI. Second, the Koelis system was replaced by 2D-TRUS system (Flex Focus 400; BK Medical, Herlev, Denmark) for routine seed implantation. For implantation, the TRUS probe was inserted into the rectum and the template was placed in contact with the perineum. A 5-mm safety margin was added and inverse planning was incorporated to enhance the implantation with correct localization. Except posterior and caudal extension, extra-prostatic extension of planning target volume up to 5 mm from prostate margin was tolerated. The vertical boundary of rectum was defined as follows; from 10 mm above to prostate base to 8 mm inferior to prostate apex. For the SIT group, 10–15 extra-numbers of radioactive seeds were implanted to the mpMRI marked intra-prostatic tumor targets.

### ADT for SIT brachytherapy

**Figure 1:**
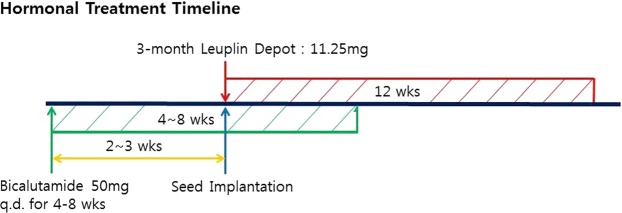
Schematic diagram of presenting ADT protocol for the SIT group.

In the current study, among the CWT group, no patient received ADT or RTx before and after implantation. However, the SIT group received peri-implantation ADT, which is the major difference compared with the CWT group. The SIT group did not undergo supplementary RTx. ADT applied to the SIT group consists of two stages. For pre-implantation therapy, bicalutamide 50 mg/day was applied for 2 to 3 weeks until one day before the operation. For post-implantation ADT, the patients of the SIT group received 3-month depot (11.25 mg) of leuprolide acetate subcutaneously on the post-implantation day-0 (approximately 5–8 hours after operation). (Fig. 1)

### Selection of treatment modality

According to the surgical modalities performed in the study, 302 patients received the CWT brachytherapy and the other 215 patients received the SIT. The CWT was performed between January 2010 and December 2014 whereas the SIT LDR-brachytherapy was performed solely from January 2015 to December 2016. Since 2015, the shift of brachytherapy technique from the CWT to the SIT has been tried for the purpose of reducing radiation induced complications. The modification of implantation technique was confirmed after thorough previewing of therapeutic effects and possible complications of the SIT by brachytherapy consortium at our institution, which consists of a medical oncologist, two urologist and two radio-oncologists.

### Follow-up

All study cohorts underwent follow-up evaluations including physical examination, PSA, International Prostate Symptom score (IPSS), simplified International Index of Erectile Function (IIEF-5), and rectal symptom questionnaire at 1-month and every 3-months post-implantation that were performed up to 36-months. CT was repeated 1-month, 3-months, 1-year and 2-years after brachytherapy. For the patients who were presented with post-implantation rectal complication ≥radiation therapy oncology group (RTOG) grade 2 such as rectal bleeding underwent sigmoidoscopy. For detection of biochemical-recurrence (BCR) after brachytherapy, we used both American Society of Therapeutic Radiology and Oncology (ASTRO) definition (the mid-point between PSA nadir and the first of 3 consecutive rises in PSA) and Phoenix definition (PSA nadir + 2 ng/mL). For the patients who were suspicious of BCR underwent 2-year post-implantation TRUS biopsy.

### Statistical analysis

All statistical evaluation of patients’ clinical outcomes were performed using SPSS package version 21.0 (SPSS Inc., Chicago, IL, USA) whereas p values < 0.05 were considered significant. Student’s t-test and Mann–Whitney test were performed to compare the basic characteristics of the study cohorts. Means and standard deviations were used for continuous variables. Categorical variables were described as proportions. To increase the validity of statistical results, we performed 1:1 propensity score matching between two surgical modality groups by using nearest neighbor matching with a maximum caliber of 0.02.The propensity score was evaluated by adjusting variables such as age, preoperative prostate-specific antigen (PSA), prostate volume, biopsy GS, and cT. After propensity matching, each study group was assigned with 190 patients. Logistic regression analyses were performed to evaluate the factors influencing post-implantation lower urinary tract symptoms (LUTS) and potency. Kaplan-Meier analyses were performed to compare biochemical-recurrence-free-survival (BRFS) differences between the CWT and SIT groups.

## Results

### Demographics and ADT

Table 1 describes the basic characteristics of the study cohort. The mean follow-up duration of the CWT and SIT groups were 89.3 months and 27.3 months, respectively. There were no significant differences between the two groups in terms of the patients’ age, initial (pre-implantation) PSA, body mass index (BMI), initial prostate volume, initial IPSS, initial IIEF-5 score and the rate of preoperative alpha-blocker use. Regarding pathologic status, no significant differences were observed between the two study groups in terms of biopsy GS, number of positive cores in prostate biopsy, and cT stages. The two groups also presented no significant differences regarding preoperative risk groups. The mean duration of ADT for the SIT group was 14.9 weeks, while the range of ADT duration was limited as 14~15 weeks to standardize the ADT period. At 3-months post-implantation, the CWT group had the mean prostate volume of 35.8cc while the pre-implantation prostate volume was 35.7cc. However, the SIT group showed profound variations regarding prostate size as their 3-months post-implantation mean prostate volume was 21.6cc while the initial volume was 34.6cc.

**Table 1:** CWT, conventional whole gland technique; SIT, sparse implantation technique; PSA, prostate specific antigen; BMI, body mass index; IIEF-5, simplified international index of erectile function; IPSS, international prostate symptoms score; ADT, androgen deprivation therapy.

A. Clinical Characteristics of Patients						
	Before matching			After matching		
	CWT§ (n = 302)	SIT (n = 215)	p value	CWT (n = 190)	SIT (n = 190)	p-value
Age,mean ± SD, year	64.9 ± 10.8	64.3 ± 11.3	0.874	64.6 ± 9.5	64.5 ± 10.4	0.951
Initial PSA, mean ± SD, ng/mL	6.8 ± 5.3	6.7 ± 4.9	0.752	6.7 ± 4.8	6.7 ± 3.2	0.970
BMI, mean ± SD, kg/m^2^	24.0 ± 2.8	23.3 ± 4.0	0.551	23.9 ± 2.1	23.8 ± 4.3	0.928
Initial prostate vol., mean ± SD, cc	35.7 ± 11.2	34.6 ± 12.8	0.439	36.0 ± 7.3	35.1 ± 8.6	0.504
Initial IIEF-5 score, mean ± SD	14.4 ± 4.6	14.2 ± 5.1	0.507	14.4 ± 4.2	14.3 ± 4.9	0.425
Initial total IPSS, mean ± SD	17.3 ± 8.2	17.6 ± 7.5	0.410	17.6 ± 8.0	17.6 ± 6.4	0.433
IPSS-voiding subscore, mean ± SD	9.7 ± 6.5	9.8 ± 8.0		10.1 ± 4.6	9.4 ± 5.7	
IPSS-storage subscore, mean ± SD	7.6 ± 3.3	8.1 ± 2.4		7.7 ± 2.9	8.3 ± 2.8	
Preoperative alpha blocker use, n (%)	245 (81.1)	171 (79.5)	0.989	153 (80.5)	154 (81.1)	0.997
Tamsulosin, n(%)	68/245 (27.8)	48/171 (28.0)		42/153 (27.5)	43/154 (27.9)	
Alfuzosin, n(%)	52/245 (21.2)	34/171 (19.9)		32/153(20.9)	32/154(20.8)	
Silodosin, n(%)	58/245 (23.7)	43/171 (25.2)		36/153 (23.5)	37/154 (24.0)	
Terazosin, n(%)	46/245 (18.8)	32/171 (18.7)		30/153 (19.6)	29/154 (18.8)	
Doxazosin, n(%)	21/245 (8.5)	14/171 (8.2)		13/153 (8.5)	13/154 (8.4)	
Biopsy GS, n (%)			0.808			0.975
3 + 3	143 (47.4)	104 (48.3)		91 (47.9)	90 (47.4)	
3 + 4	111 (36.7)	78 (36.3)		70 (36.8)	72 (37.8)	
4 + 3	48 (15.9)	33 (15.4)		29 (15.3)	28 (14.7)	
≥4 + 4	0 (0.0)	0 (0.0)		0 (0.0)	0 (0.0)	
Positive cores at biopsy, n (%)			0.882			0.866
1	101 (33.4)	67 (31.2)		64 (33.7)	62 (32.6)	
2–3	156 (51.7)	120 (55.8)		99 (52.1)	101 (51.2)	
≥4	45 (14.9)	28 (13.0)		27 (14.2)	27 (14.2)	
Clinical tumor stage, n (%)			0.561			0.991
cT1	128 (42.4)	95 (44.2)		81 (42.6)	82 (43.2)	
cT2a	105 (34.8)	76 (35.3)		66 (34.7)	67 (35.3)	
cT2b	67 (22.2)	43 (20.0)		42 (22.1)	40 (21.0)	
cT2c	2 (0.7)	1 (0.5)		1 (0.5)	1 (0.5)	
≥cT3a	0 (0.0)	0 (0.0)		0 (0.0)	0 (0.0)	
Preoperative risk group			0.889			0.758
Low	140 (46.4)	101 (47.0)		88 (46.3)	91 (47.9)	
Intermediate	162 (53.6)	114 (53.0)		102 (53.7)	99 (52.1)	
High	0 (0.0)	0 (0.0)		0 (0.0)	0 (0.0)	
**B. Post-implantation potency and voiding function**						
Mean duration of ADT, mean ± SD, weeks	·	14.9 ± 0.3		·	14.8 ± 0.4	
3-months post-implantation prostate vol., mean ± SD, cc	35.8 ± 12.7	21.6 ± 6.9	**0.031**	35.9 ± 10.5	21.0 ± 3.1	**0.029**
Prostate vol. reduction after ADT						
<10	·	25 (11.6)		·	22 (11.6)	
10 ~ < 20	·	42 (19.5)		·	38 (20.0)	
20 ~ < 30	·	88 (40.9)		·	79 (41.6)	
30 ~ < 40	·	50 (23.3)		·	44 (23.1)	
40 ~ < 50	·	10 (4.7)		·	7 (3.7)	
3-months post-implantation IIEF-5, mean ± SD	11.2 ± 3.8	9.6 ± 3.1	0.089	11.3 ± 3.2	9.4 ± 2.7	0.075
3-months post-implantation total IPSS, mean ± SD	19.2 ± 7.5	15.9 ± 6.7	**0.038**	19.1 ± 5.9	15.9 ± 5.5	**0.043**
IPSS-voiding subscore, mean ± SD	11.2 ± 4.0	8.5 ± 5.3		11.8 ± 5.1	8.2 ± 4.8	
IPSS-storage subscore, mean ± SD	8.3. ± 3.6	7.4 ± 3.2		7.9. ± 4.4	7.1 ± 4.2	
12-months post-implantation IIEF-5, mean ± SD	12.5 ± 4.8	13.8 ± 6.1	0.103	12.9 ± 4.0	13.6 ± 5.2	0.092
12-months post-implantation total IPSS, mean ± SD	17.6 ± 9.4	17.8 ± 7.7	0.313	17.6 ± 6.5	17.7 ± 7.0	0.398

^§^No patient in the CWT group underwent ADT before and after implantation.

^Ŧ^Low-dose-sildenafil: 25 mg/day (daily dose).

Among the SIT group, most patients (88 patients, 40.9%) had their prostate volume reduction between 20–30% after ADT, and the second most patients (50 patients, 23.3%) achieved 30–40% of prostate volume reduction (Table 1). Moreover, of patients with a pre-implantation prostate size of greater than 40cc, majority of patients (93.7%) reached a prostate volume of ≤40 cc after ADT. Regarding LUTS at 3-months post-implantation, the SIT group presented significantly better IPSS than the CWT group (p = 0.038). Furthermore, the CWT showed an increase of mean total IPSS (pre-implantation: 17.3, post-implantation: 19.2), but the SIT group had a decrease in mean total IPSS (pre-implantation: 17.6, post-implantation: 15.9). The SIT group showed a greater decrease in mean IIEF-5 score (9.6) compared to the CWT group (11.2) at 3-months post-implantation, but the difference was not statistically significant (p = 0.089). To increase the statistical reliability, we performed the propensity matching between the two groups and each group was assigned with 190 patients after the matching (Table 1). The post-matching results were not significantly different from the pre-matching data, but some 3-months post-implantation values including prostate volume and mean IIEF-5 demonstrated greater difference between the two groups after propensity matching. At 12-months post-implantation, both study groups recovered their erectile function near to the baseline level whereas the mean IIEF-5 score reached over 85% of the pre-implantation scores (CWT: 12.5, SIT: 13.8, p = 0.103). In both study groups, the mean total IPSS score decreased down to the baseline level 12-months after brachytherapy.

### Post-implantation dosimetry, complications and oncological outcomes

The dose parameters of both study groups, regarding prostate, urethra, and rectum are described in Table 2. The immediate post-implantation dosimetry shows that the D90 (minimum prescription dose covering 90% of prostate volume) of the CWT group (173 Gy) was significantly higher than 157.5 Gy of SIT group (p = 0.012). The CWT group also incorporated a greater number of implanted seeds (range: 75–83) than the SIT group (range: 50–61). For the urethra, the CWT showed initial VU150 (fractional volume of the urethra receiving 150% of the prescribed dose) of 15.7%, which was greater than 10.6% of the SIT group. No significant differences between the two groups were observed in terms of VR150 (volume of the rectum receiving 150% of the prescribed dose) for the rectum. At 30-day post-implantation, no significant dosimetric differences were observed between two groups regarding D90 and VR150. For urethra, the SIT group showed a significantly lower value of VU150 (10.9%) than the CWT group (16.1%) (p = 0.011).

**Table 2:** Post-implantation dosimetry and pathologic findings.

	CWT (n = 302)	SIT (n = 215)	p-value
At implantation			
D90, mean (range), Gy	173 (170–185)	157.5 (154–163)	0.012
VU150, mean (range), %	15.3 (12.2–17.5)	9.8 (7.4–10.8)	0.001
VR150, mean (range), cc	0.2 (0–1.3)	0.1 (0–0.5)	0.910
Number of implanted seeds, mean (range)	80 (75–83)	55 (50–61)	0.025
30-day post-implantation			
D90, mean (range), Gy	156 (147–163)	152 (145–156)	0.903
VU150, mean (range), %	16.1 (15.2–19.8)	10.9 (9.1–14.1)	0.011
VR150, mean (range), cc	0.5 (0.2–2.0)	0.2 (0.1–0.7)	0.067
Post-implantation complications (3-months)			
Urinary toxicity ≥ RTOG grade 2, n (%)	1 (0.3)	0 (0.0)	0.399
Rectal toxicity ≥ RTOG2 grade 2, n (%)	3 (1.3)	0 (0.0)	0.143
Post-implantation prostate biopsy (2-year)			
Number of performed biopsy cases, n	5	3	
Patients with positive cores in post-implantation biopsy, n	0	0	

Mean duration of ADT = 14.9 weeks (range 14–15 weeks).

CWT, conventional whole gland technique; SIT, sparse implantation technique; D90, minimum prescription dose covering 90% of prostate volume; VU150, fractional volume of the urethra receiving 150% of the prescribed dose; VR150, volume of the rectum receiving 150% of the prescribed dose; RTOG, radiation therapy oncology group.

**Figure 2:**
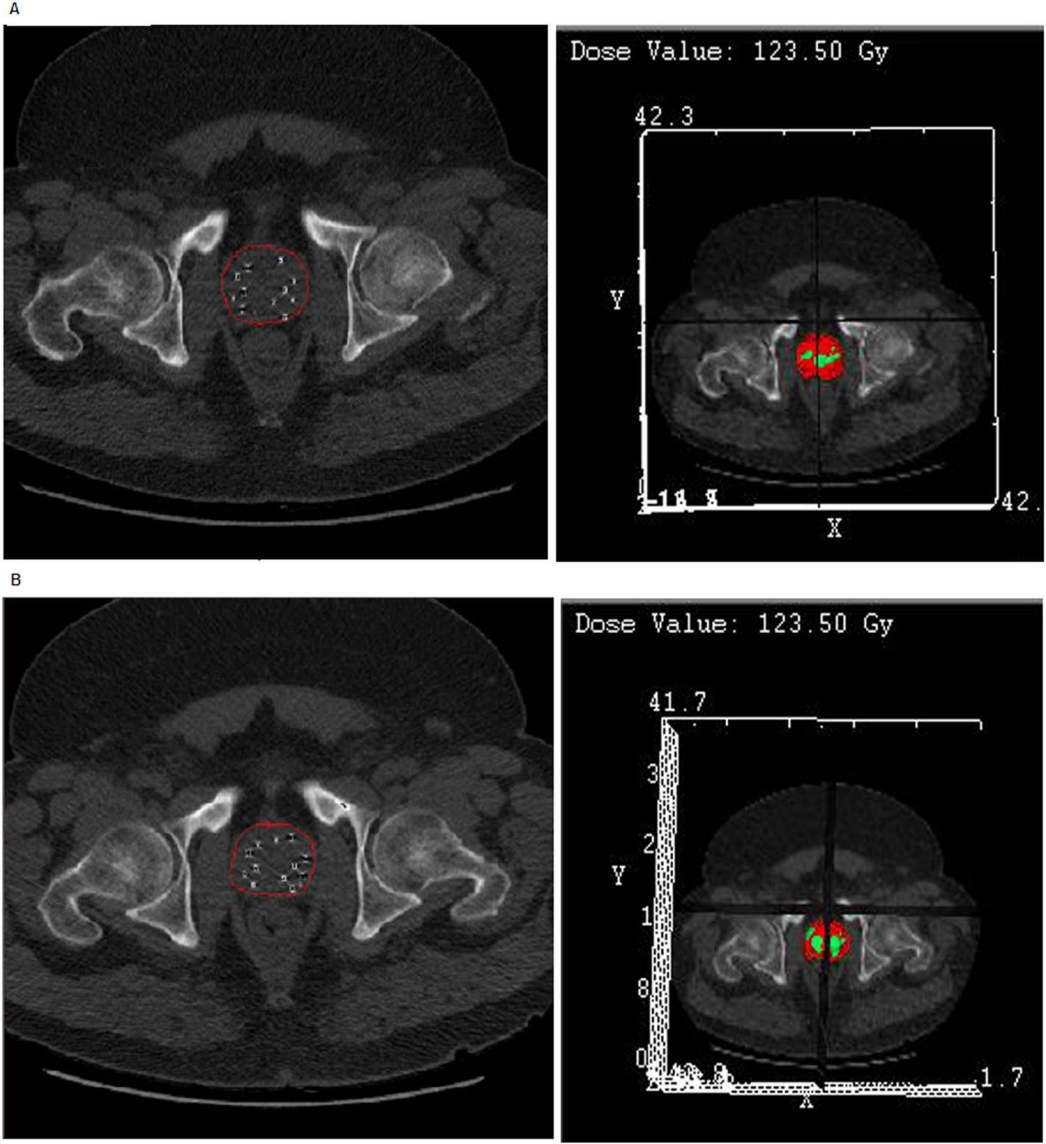
Prostate dosimetry of a patient in the SIT group; (**A**) immediate after implantation, (**B**) 3-months post-implantation, the patient had 25% of prostate volume reduction after ADT and inter-seed spacing became smaller as prostate sized decreased (seed migration within prostate gland).

**Figure 3:**
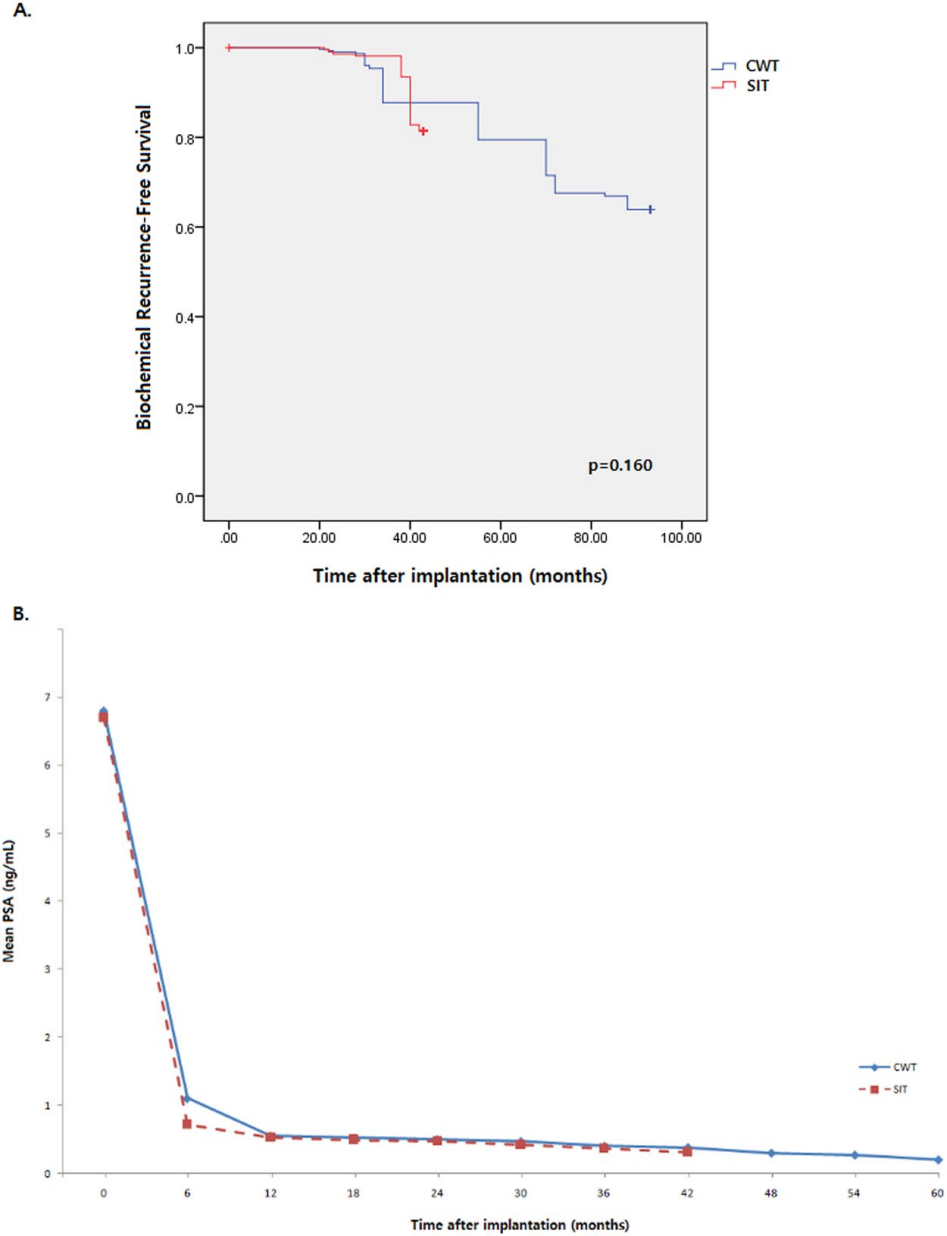
(**A**) Kaplan-Meier analyses for BRFS evaluation were performed. No significant differences regarding BRFS were observed between the two groups. (**3B**) Post-implantation PSA variations were plotted and both study groups reached PSA level ≤ 1.0 ng/mL within 12-months post-brachytherapy.

Figure 2 presents the pelvic CT and dosimetry of a patient in the SIT group, which was performed immediately after implantation (Fig. 2A) and 3-months post-implantation (Fig. 2B). According to the result, ADT induced a prostate volume reduction by 25% at 3-months post-implantation and inter-seed spacing became smaller. Regarding 3-months post-implantation complications, no statistically significant differences were observed between the two groups, but the CWT group had 1 patient with urinary toxicity ≥RTOG grade 2 and 3 patients with rectal toxicity ≥RTOG grade 2 whereas the SIT group did not present any case of urinary or rectal complications ≥grade 2. At 2-year post-implantation, 5 patients from the SIT group, who were categorized as preoperative intermediate-risk, underwent 2-year post-implantation prostate biopsy due to three consecutive PSA increase indicating BCR, and all of these patients were pathologically negative for persistent malignancy in their 2-year follow-up biopsy. The patients had a gradual decline of PSA without additional treatment and consequently reached PSA nadir. Among the CWT group, 3 patients (one patient: preoperative low-risk, two patients: preoperative intermediate-risk) had BCR with three consecutive rise of PSA level at 2-year post-implantation. The patients received 2-year post-implantation prostate biopsy and benign inflammatory prostatic tissues were confirmed through the corresponding biopsy.

According to logistic regression analysis results (Table 3), post-implantation LUTS were significantly associated with the CWT group (p = 0.023). Furthermore, Kaplan-Meier analyses (Fig. 3A) were performed and the results presented that no significant differences regarding BRFS were observed between the two groups (p = 0.160). Regarding the mean PSA variations after brachytherapy (Fig. 3B), the SIT group showed more rapid drop of the mean PSA (0.72 ng/ml) at 6-months post-implantation than the CWT group (1.1 mg/ml), and both groups reached levels of PSA ≤ 1.0 ng/mL within 12-months post-brachytherapy. The mean PSA levels persistently decreased to levels of PSA ≤ 0.5 ng/mL at 48-months post-implantation in both study groups.

**Table 3:** After matching multivariate analysis of post-implantation LUTS predictors

Factors	OR	95% CI	p-value
Age	1.523	0.687–5.388	0.430
Initial PSA	0.368	0.102–4.497	0.711
Initial prostate volume	0.673	0.405–1.125	0.136
Surgical modality			
ST	Reference		
CWT	3.810	1.332–7.641	0.023
Clinical stage	1.101	0.801–1.205	0.870

LUTS, lower urinary tract symptoms; PSA, prostate specific antigen; ST, sparse technique; CWT, conventional whole gland technique.

## Discussion

**Figure 4:**
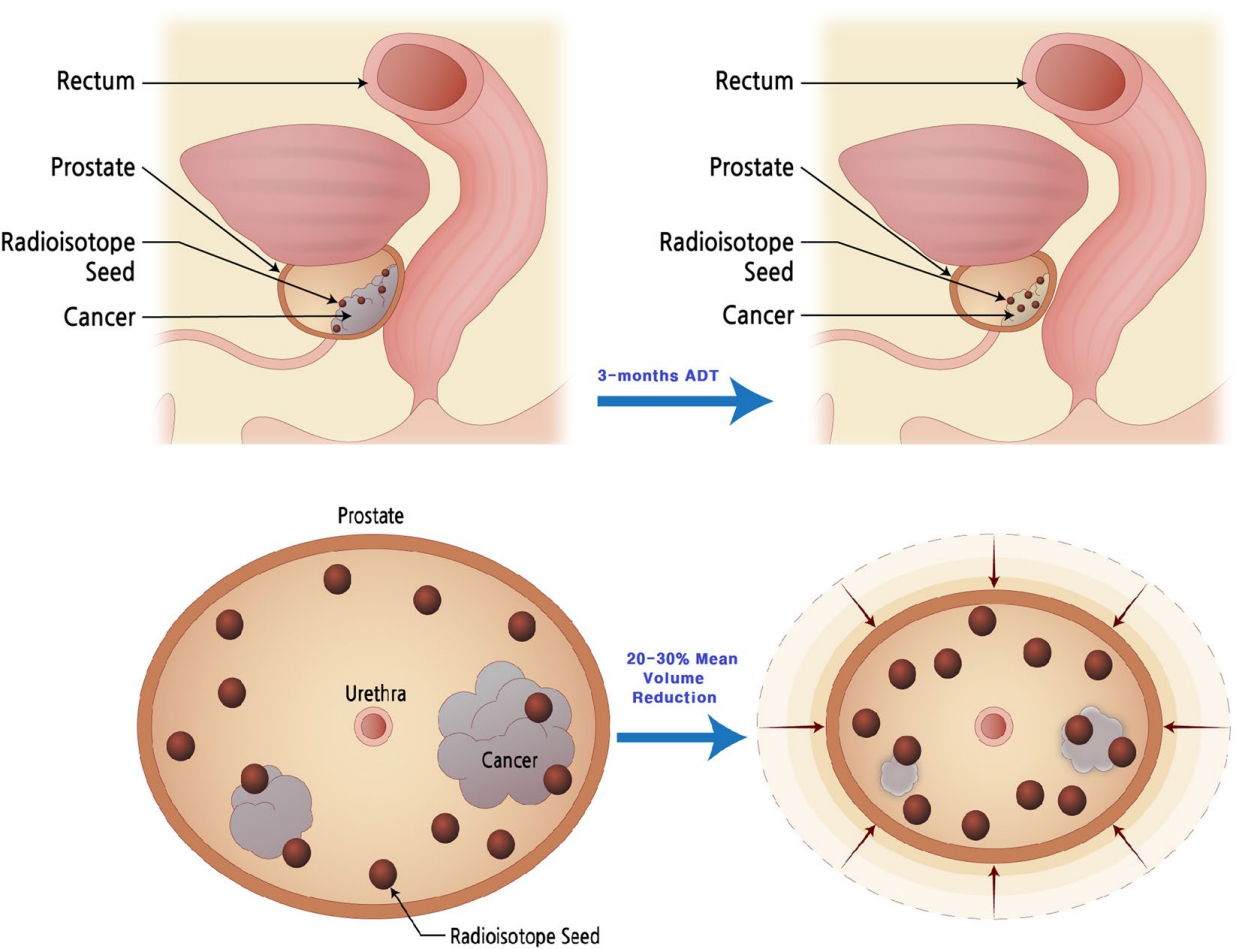
The method of the sparse implantation technique brachytherapy.

As LDR-brachytherapy for the treatment of localized PCa has been performed worldwide, the application of ADT for the purpose of prostate size reduction and additional PCa treatment has been emphasized^7^.To our knowledge, there has been no standardized protocol for ADT in combination with brachytherapy, and the ADT is generally performed to the patients with a large pre-implantation prostate volume^7,10^. Frazier *et al*.^10^ performed trasperineal prostate implantation with ADT to the patients with initial prostate volume ≥50cc and the median reduction of prostate volume after 3-months ADT was 29%. They also described that the patients treated solely with leuprolide were not different with leuprolide plus antiandrogen treated patients regarding prostate volume reduction. Our study results were similar to these previous studies as ADT induced 40.9% and 23.3% of the SIT group patients undergoing 20–30% and 30–40% of prostate volume reduction, respectively. Moreover, evaluating the actual dose of irradiation in LDR-brachytherapy is often technically challenging because of prostate edema induced by implantation^10,11^. Therefore, this study aimed to standardize the ADT use in LDR-brachytherapy for the purpose of prostate malignancy control as well as post-implantation complications including LUTS induced by prostate edema and irradiation. At the beginning of the study, we speculated that prostate volume reduction by ADT would allow a decrease of initial implantation prescription dose as described in Fig. 4. In accordance with the idea, we also extrapolated that the initial prescription dose reduced by a certain degree would subsequently decrease post-implantation complications while maintaining acceptable dosimetric and oncological outcomes, since the target volume of prostate would gradually decrease due to concurrent ADT. Furthermore, several previous studies suggested synergy between RTx and concurrent ADT as ADT promotes apoptosis of PCa cell by decreasing malignant cell hypoxia and DNA repair^12^. Based on those studies, we assumed the concurrent ADT with brachytherapy might derive outstanding oncological outcomes for early localized PCa. Thus, we employed radioactivity decay curve and I-125 seed nomogram of prostate volume, which derived 123.5 Gy for the optimal prescription dose for the SIT group. The current study is the first study evaluating the feasibility of 123.5 Gy prescription dose whole gland LDL-brachytherapy in combination with ADT. In this study, we actively incorporated the technical advantages of mpMRI for outlining and focusing PCa suspicious targets that enabled extra-seed implantation to the target volume within prostate gland. Among the SIT group of this study, we implanted 15 seeds for every 3.3cc of mpMRI marked target volume and this method is similar to the previous focal therapy studies, including Brun*et al*.^13^. According to their ultra-focal brachytherapy study, Brun and the colleagues implanted 14 to 32 seeds for the mean target volume of 3.33cc based on the phantom study. Thus, we believe the SIT of brachytherapy can be interpreted as a new implantation modality employing the advantages of both whole gland and focal implantation techniques. With the SIT, both compatible oncological outcomes to the CWT and relatively smaller number of post-implantation complications could be achieved within low- and intermediate-risk PCa patients.

Most patients experience some degree of LUTS after brachytherapy and 2–22% of the brachytherapy patients present with acute urinary retention^14,15^. Although some previous studies reported no correlation between IPSS elevation and total implant activity^13^, other studies, including Wallner *et al*., suggested the correlation between post-implantation LUTS and radiation dose to urethra^16,17^. Waller *et al*.^17^ described that the maximum urethral dose ≥250% of the minimum peripheral dose is associated with urinary complications. In the current study, almost 80% of each study group received various types of alpha-blockers treatment before brachytherapy. After implantation, both groups were treated with only tamsulosin 0.4 mg plus sildenafil 25 mg combination therapy, which was proven effective for managing post-implantation urinary and sexual morbidities in our previous study^18^. Although both groups received the same therapy for managing post-implantation LUTS, the SIT group showed profoundly better IPSS than the CWT group. Notably, the 3-months post-implantation mean total IPSS of the SIT group was even lower than the pre-implantation values. Furthermore, the logistic regression analysis results showed that the CWT group favors post-impanation LUTS than the SIT group. We interpreted this significant early improvement of urinary symptoms within the SIT group as the results from ADT and relatively lower urethral radiation exposure. Moreover, the mean total IPSS score reached to the baseline level in both implantation modalities at 12-months post-brachytherapy, indicating well tolerable therapeutic effect of tamsulosin 0.4 mg plus sildenafil 25 mg for a management of post-implantation LUTS.

Regarding post-implantation potency, the CWT showed higher mean IIEF-5 score compared to the SIT at 3-months post-brachytherapy, although the difference was statistically insignificant. However, the SIT group had a slightly higher mean IIEF-5 score than the CWT group at 12-months post-brachytherapy. According to some previous studies, various types of sexual symptoms including erectile dysfunction (ED) occur after brachytherapy^19^. Merrick *et al*.^19^ reported 6–90% of patients experience post-implantation ED. They also described that post-implantation ED is multifactorial process, but radiation dose to the bulb of penis was significantly related to ED. Other studies suggested that pre-implantation potency is the most important predictor of post-implantation ED^20,21^. In this study, both groups had no significant differences regarding pre-implantation potency. According to our study results, we postulated that ADT might be more strongly associated with short-term post-implantation potency than the reduced prescription dose. However, the corresponding results also suggest that the long-term recovery of potency after brachytherapy is mainly dependent on the initial prescription dose or the number of implanted radioactive seeds.

Although our study results showed that the CWT group had a greater urethral radiation exposure in both pre- and post-implantation (30-day) dosimetry, no significant differences were observed regarding post-implantation urinary toxicity ≥RTOG 2 between the two groups. These findings derived two interesting postulations as follows. First, the urethral dose and total implant activity are potential risk factors of urinary symptoms after brachytherapy. Second, even if a patient experiences LUTS after brachytherapy, the risk of moderate to severe post-implantation urinary toxicity to occur might be very low within the range of conventionally used implantation dose as Merrick *et al*.^14^ suggested. Regarding rectal complications, only the CWT group showed rectal toxicity ≥RTOG 2, but all 3 corresponding patients with rectal toxicity showed grade 3 internal hemorrhoid confirmed by sigmoidoscopy. Thus, the effect of the reduced prescription dose of the SIT group on rectal complications should be further investigated in the future study.

During post-implantation PSA follow-up, 3 patients from the CWT group and 5 patients from the SIT group satisfied the definition of BCR, and they underwent 2-year post-implantation prostate biopsy. All of these patients had benign prostate tissues confirmed by pathologic evaluations. Although we used both ASTRO and Phoenix definitions to define BCR, confirming BCR was still controversial and technically challenging as many previous studies described^22^. Moreover, some studies demonstrated that sometimes the PSA nadir after brachytherapy can be defined only after the PSA rise^22,23^. Thus, post-implantation PSA spike pattern and follow-up prostate biopsy within the SIT group should be further researched through upcoming study with a greater size cohort and longer follow-up.

Regarding oncological outcomes after brachytherapy, Stock *et al*.^24^ presented the correlation between the prescription dose and the freedom-from-PSA-failure (FFPF). In addition, Wallner and colleagues showed that 12-year FFPF was 87% and 79% for the low-risk and intermediate-risk groups, respectively^25^. In the current study, there were no significant differences regarding PCSS between the two groups, implying 123.5 Gy of reduced implantation dose might be tolerable for the low- and intermediate-risk PCa treatment. However, for general acceptance of the SIT with concurrent ADT, the PCa control safety of the SIT needs to be further evaluated.

Although we tried to minimize the negative impact of imprecise procedure, this study carries some conceptual and technical limitations. First, there are some degrees of inter-personal differences regarding prostate volume reduction by ADT. Due to this volume reduction differences, using a single modified implantation dose may not be suitable for every PCa patient. Moreover, the follow-up period for some patients of the SIT group is relatively too short to analyze the oncologic outcomes. Second, the post-implantation prostate size of the cohort was measured by three different radiologists, and this could contribute some negative effects of measurement variability to the study. Third, this study used only a single type of ADT modality, but other ADT methods need to be analyzed to establish the optimal ADT protocol for the sparse implantation technique. For the last, no randomization of the cohort was performed, which could be a potential weakness of this study.

Although there are some limitations and its retrospective nature, the current study is still the first study evaluating the feasibility of the reduced implantation dose brachytherapy with concurrent ADT use.

## Conclusions

The present study has suggested the first concept of the reduced implantation dose brachytherapy in concurrent use of ADT. However, for the new SIT LDR-brachytherapy to be accepted for general use, further evaluations are required to establish the optimal reduced implantation dose. The timing (concurrent or neoadjuvant) and duration of ADT also need to be further researched through the upcoming studies.
